# Optical, Electrical and Thermal Properties of Organic–Inorganic Hybrids with Conjugated Polymers Based on POSS Having Heterogeneous Substituents

**DOI:** 10.3390/polym11010044

**Published:** 2018-12-29

**Authors:** Kazunari Ueda, Kazuo Tanaka, Yoshiki Chujo

**Affiliations:** 1 Department of Polymer Chemistry, Graduate School of Engineering, Kyoto University, Katsura, Nishikyo-ku, Kyoto 615-8510, Japan; rouge_asteroide_agile_tigre_53@yahoo.co.jp (K.U.); chujo@poly.synchem.kyoto-u.ac.jp (Y.C.); 2 Matsumoto Yushi-Seiyaku Co., Ltd., 2-1-3, Shibukawa-cho, Yao-City, Osaka 581-0075, Japan

**Keywords:** POSS, hybrid, carrier mobility, thermal stability, conjugated polymer

## Abstract

Preparation of organic–inorganic hybrids with conventional conjugated polymers such as polyfluorene (PF) and poly(3-hexylthiophene) (P3HT) were demonstrated via the facile blending in solution by employing polyhedral oligomeric silsesquioxane (POSS) having heterogeneous alkyl substituents. From the optical measurements, it was shown that the modified POSS derivatives played a critical role in facilitating amorphous state of polymer matrices. Interestingly, although inter-strand interaction decreased after POSS addition in the hybrid films, thermal stability can be enhanced in the presence of the modified POSS with long alkyl chains. Furthermore, it was demonstrated that carrier mobilities through the hybrid film was minimally reduced by POSS. These results suggest that POSS should be a versatile building block to form hybrid with various types of polymers for enhancing durability without loss of electronic properties of organic components.

## 1. Introduction

By mixing organic molecules and inorganics at the nano level, robust materials, called organic–inorganic hybrids, are created, which have multiple functions originating from both components [1]. Because of their high durability, hybrids are regarded as a platform for realizing advanced optoelectronic materials containing conjugated molecules and polymers. For example, electric-conductive hybrids are obtained by introducing conductive organic crystals into a polymer hybrid matrix based on silica [2]. In particular, the resulting hybrids show a waterproof character and higher heat resistance than that of the pristine organic crystal [2]. By loading a series of conjugated molecules onto hybrid matrices, multiple optical properties are readily expressed. Intense white-light luminescence is observed from dye-loaded robust hybrids [3]. Thus, hybrid formation is currently recognized as a valid strategy for enhancing durability of organic products including conjugated polymers. However, hybrid formation is usually performed via the sol-gel reaction in polar solvents with acid or base catalysts [4,5,6]. Thereby, aggregation followed by generation of inhomogeneity and degradation of conjugated polymers is often induced during the sol–gel reactions. Furthermore, carrier-transport ability could decrease after hybrid formation compared to that of the pristine organic material due to intrinsic high electric resistance of silicate. Thus, our next challenge is not only to establish the facile manner for hybrid formation without critical losses of electric properties of conjugated polymers but also to systematically study the electric properties of the polymers inside hybrids.

Polyhedral oligomeric silsesquioxane (POSS) has attracted attention for preparing functional hybrids [7,8,9,10] and thermal stability of conjugated molecules that are conventionally used as a key components in modern organic electric devices can be improved simply by chemically connecting to POSS [11,12,13,14,15,16]. Recently, POSS is also regarded as a platform for designing molecular fillers that add desired functions in polymers [17,18,19,20,21]. By modulating the organic substituents at the vertices of the cage, compatibility with various media can be adjusted [22,23,24,25,26,27,28]. Indeed, introduction of POSS derivatives into conventional polymers is shown to significantly improve the thermal stability and mechanical properties, with or without covalent bonds [29,30,31,32,33]. Moreover, POSS has extremely-high compatibility with conjugated polymers [34]. Homogeneous films with higher concentrations of POSS (>40 wt %) can be prepared via simple mixing in solution. Although most POSS containing rigid or short R groups crucially decrease thermal degradation temperatures by 100 °C due to suppression of inter-strand stacking by the cage, we found that longer alkyl-modified POSS were able to reinforce thermal stability of the matrices [34]. From these observations, we propose that, if POSS derivatives are homogeneously dispersed in the matrix, it can be said that a similar situation to the typical hybrids should be created. In other words, we suggest that POSS can be a versatile “element-block” [35,36], which is the minimum functional unit composed of heteroatoms for preparing “designable hybrids” without sol–gel methods. Previously, optical and thermal properties were investigated, however electrical properties were not examined. To practically use POSS-based hybrids as an optoelectronic materials, influence of the POSS addition on electrical properties of conjugated polymers is essential.

Herein, we report the influence on electronic properties and enhanced thermal stability of hybrids composed of conjugated polymers by loading POSS derivatives having dual types of alkyl substituents. Three types of heterogeneous POSS derivatives with two kinds of alkyl substituents were synthesized, and. from the solution method, polymer hybrids with conventional conjugated polymers such as polyfluorene (PF) and poly(3-hexylthiophene) (P3HT) were prepared by loading octa-substituted alkyl POSS. The series of measurements for optical and thermal properties and carrier-transport ability revealed that the heterogeneous POSS derivatives can improve thermal stability without critical losses of other properties. This is the first example, to the best of our knowledge, demonstrating the applicability of POSS “element-block” for fabricating hybrids with conjugated materials without losses of optoelectronic properties of conjugated polymers.

## 2. Results and Discussion

**Scheme 1:**
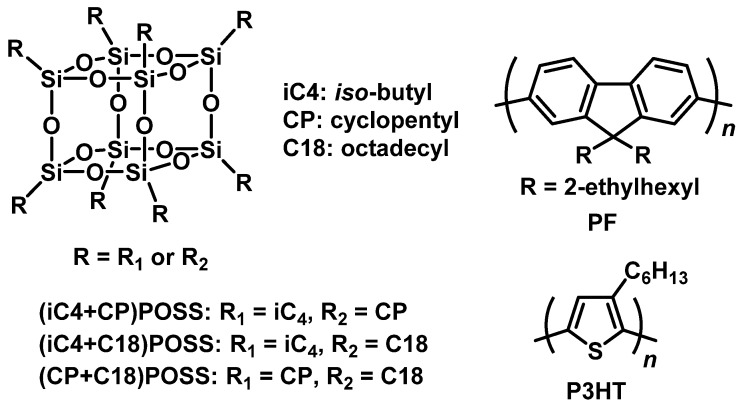
Chemical structures of the modified POSS and conjugated polymers used in this study.

The chemical structures of the materials used in this study are shown in Scheme 1. It was shown from the study with octa-substituted POSS and conjugated polymers that the *iso*-butyl (iC4) and cyclopentyl (CP) groups on POSS have relatively lower affinity toward conjugated polymers and induced loss of thermal stability, while the octadecyl (C18) group can enhance thermal stability of the matrices [34]. It was assumed that entanglement with polymer chains could disturb molecular motions followed by thermal degradation. From these three alkyl groups, two substituents were introduced into POSS. The synthesis of a series of heterogeneous POSS derivatives was performed with the previously established protocol [25]. It was known that the products used in further analyses included cage mixtures, which are structural isomers and comprise molecular-weight distributions originating from variation of the molar ratios of the two alkyl silane starting materials.

PF and P3HT are conventionally used as a carrier-transport layer in organic light-emitting devices and as a traditional donor–acceptor bulk-heterojunction active layer in polymer photovoltaic cells, respectively. Therefore, enhancement of thermal stability is of great importance for extending device lifetimes. PF and P3HT were prepared via the Yamamoto and oxidation coupling reaction according to the previous methods, respectively (Table S1) [37,38,39].

The polymer films containing POSS derivatives were prepared using solution blending of each POSS and polymer after drying on the quartz substrate. For evaluating carrier migration ability, the films were prepared on the ITO electrode via the spin-coating.

In Figure S1, the appearances of the hybrid films are presented. Accordingly, at loading levels of up to 40 wt % POSS, significant turbidity and phase separation were not observed in the films. The transparency resulted from compatibility that eliminated heterogeneous POSS crystallization. Heterogeneity and loss of transparency was observed in control samples containing two types of octa-substituted POSS. Additionally, phase separation and inhomogeneity were hardly detected in nano level in the scanning electron microscopic (SEM) observations (Figure S2). These data represent that POSS fillers can be dispersed in conjugated polymer films with high homogeneity. Thus, it can be said that hybrid materials were obtained with the POSS “element-blocks” without sol–gel reactions [25,34]. Further experiments were performed with the samples containing 40 wt % POSS.

Initially, light-absorption properties were investigated with the hybrid films (Table 1 and Figures S3–S6). In the PF film, it was known that aggregation at partial polymer chains induces red-shift of the absorption band [34]. In the previous report, it was demonstrated that POSS derivatives were able to facilitate formation of amorphous state followed by blue-shifted absorption band [34]. The steric structure of the silica cube could play a critical role in minimizing the crystallization of polymer chains. The spectra indicate that such an effect was realized. By loading POSS into the PF matrices, the absorption bands around 400 nm were sharpened and the apparent absorption maxima were shifted to shorter wavelength region. These data support that POSS derivatives induced an amorphous state within the PF matrices. In contrast, larger degrees of peak shifts were observed from heterogeneous POSS than those from the mixtures with two types of octa-substituted POSS. Because of larger miscibility of heterogeneous POSS than octa-substituted POSS, amorphous state could be effectively created by heterogeneous POSS.

It was observed in the previous report that red-shift of the absorption bands were induced by adding POSS to the P3HT film (Figures S3–S6) [34]. It was proposed that polymer chains can form favorable conformation for extending conjugation lengths through the polymer main-chains by releasing structural restrictions caused by inter-chain interaction. In this study, same tendencies were observed. By adding POSS, peak shifts to longer wavelength region were observed, indicating that POSS molecules also enhanced homogeneity in the P3HT film. In the P3HT hybrid, undesired elevation of background and peak broadening were hardly induced by POSS. These data support that homogeneous state can be obtained by POSS.

Luminescent properties were also examined with the hybrid films (Table 1 and Figures S7–S10). In the PF matrices, significant effects were slightly observed in the spectra and emission efficiencies by adding POSS. In contrast, large degrees of red-shift were detected in the emission spectra of the P3HT hybrids. Similar to electronic structures in the ground state, main-chain conjugation should be elongated by POSS. In summary, the influence of the heterogeneous POSS addition on optical properties was largely similar to those of octa-substituted POSS. It was shown that POSS derivatives played a role in improving homogeneity of the polymer matrices.

**Figure 1:**
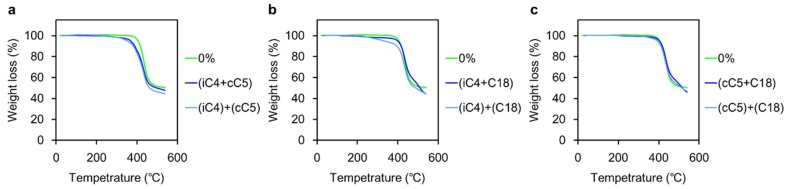
TGA profiles of the PF hybrids in the absence and presence of: (**a**) 40 wt % (iC4 + CP)POSS and the mixture with 20 wt % iC4-POSS and 20 wt % CP-POSS; (**b**) 40 wt % (iC4 + C18)POSS and the mixture with 20 wt % iC4-POSS and 20 wt % C18-POSS; and (**c**) 40 wt % (CP + C18)POSS and the mixture with 20 wt % CP-POSS and 20 wt % C18-POSS.

**Figure 2:**
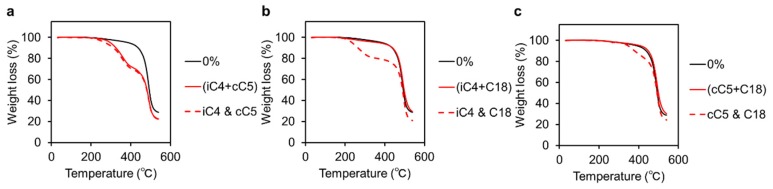
TGA profiles of the P3HT hybrids in the absence and presence of: (**a**) 40 wt % (iC4 + CP)POSS and the mixture with 20 wt % iC4-POSS and 20 wt % CP-POSS; (**b**) 40 wt % (iC4 + C18)POSS and the mixture with 20 wt % iC4-POSS and 20 wt % C18-POSS; and (**c**) 40 wt % (CP + C18)POSS and the mixture with 20 wt % CP-POSS and 20 wt % C18-POSS.

Next, thermal stability of the hybrid films was evaluated with thermogravimetric analysis (TGA, Figure 1 and Figure 2). Degradation temperatures are listed in Table 2. According to the previous report on the PF and P3HT hybrids with octa-substituted POSS, critical decreases in degradation temperatures were caused by adding most of octa-substituted POSS [34]. It is likely that thermal motions were induced due to disruption of inter-chain interactions by the rigid POSS cages. In this study, the thermal stabilities of the hybrids with both polymers were maintained even up to 40 wt % loadings of the heterogeneous POSS. The (iC4 + CP)POSS was the lone exception to this trend as it induced a significant reduction of thermal stability. It is suggested that entanglement with the dodecyl group and polymer side chains contributed to suppressing molecular motions of polymer chains. Consequently, critical loss of thermal stability could be avoided.

Finally, the electric properties of hybrid materials were examined. The carrier mobilities of the hybrid films were evaluated by the time-of-flying (TOF) experiment in which the transient photocurrent generated with a laser pulse was monitored by an oscilloscope (Table S4) [40,41,42]. Figure S11 shows the representative device appearances for TOF measurements with PF and P3HT hybrids. The carrier mobilities in the hybrid films were measured to be in the order of 10^−3^ cm^2^ V^−1^ s^−1^. Compared to each pristine polymer, significant changes in the mobility were not observed. Furthermore, the carrier mobilities decreased with an increasing external electrical field with similar extents (Figures S12 and S13). Typically, additives in polymer matrices create carrier traps, followed by critical decreases in carrier mobilities; however, it was revealed that POSS had minimal influence on carrier transport processes through both polymer matrices. According to the previous reports on the conductive composites containing mixed-valence tetrathiafulvalene nanowires, conductivity can be maintained in the bulk materials by gathering nanowires at the surface even in the insulating matrices [43,44,45,46]. In this study, although POSS could hardly work as a carrier transporter, charges could smoothly pass through homogeneous polymer matrices. This finding and our data demonstrate that hybrid formation can be accomplished without changes in electric properties of conjugated polymers by employing POSS “element-blocks”.

## 3. Conclusions

It was demonstrated that POSS “element-blocks” enable transformation of conjugated polymers into thermally-stable hybrid materials without critical losses of optical and electronic properties provided by the organic components. This was accomplished by POSS cages controlling the solid-state packing within the hybrid composition. Moreover, by selecting the type of substituents as the longer alkyl chain on POSS, miscibility was enhanced, indicating that hybrid formation based on POSS “element-blocks” possess a wide applicability to various polymer matrices. This information on property–structure relationships would be helpful for designing further effective fillers for modulating optoelectronic functions as well as thermal properties of conjugated polymers. In the development of organic opto-electronic devices, reinforcement of durability is still one of big issues to be overcome. Our concept and materials could be valid to satisfy these demands.

## Figures and Tables

**Table 1:** Optical property changes by hybrid formation with POSS *^a^.*

POSS filler	PF			P3HT		
	Δ*λ*_abs_ (nm) *^b^*	Δ*λ*_em_ (nm) *^c^*	*Φ*_PL_ (%) *^d,e^*	Δ*λ*_abs_ (nm) *^f^*	Δ*λ*_em_ (nm) *^g^*	*Φ*_PL_ (%) *^e,h^*
(iC4 + CP)	−16	+3	+3	+22	+20	+1.1
(iC4 + C18)	−21	+3	+6	+26	+30	+1.5
(CP + C18)	−15	+3	+6	+27	+24	+1.4
iC4&CP	−16	+4	+6	+24	+27	+1.3
iC4&C18	−11	+2	+6	+24	+27	+1.5
CP&C18	−8	+1	+6	+24	+25	+1.7

*^a^* 40 wt % heterogeneous POSS and mixture with 20 wt % each POSS. *^b^* Determined with the peak at 407 nm. *^c^* Determined with the peak at 509 nm (*λ*_ex_ = 407 nm). *^d^* Compared with 41% from the pristine polymer. *^e^* Determined as an absolute value with the integration sphere method. *^f^* Determined with the peak at 484 nm. *^g^* Determined with the peak at 639 nm (*λ*_ex_ = 484 nm). *^h^* Determined with 0.7% from the pristine polymer.

**Table 2:** Thermal property changes by hybrid formation with POSS*^a^*.

POSS filler	PF *^a^*		P3HT *^b^*		POSS	
	Δ*T*_d5_ (°C) *^b^*	Δ*T*_d20_ (°C) *^c^*	Δ*T*_d5_ (°C) *^d^*	Δ*T*_d20_ (°C) *^e^*	*T*_d5_ (°C) *^d^*	*T*_d20_ (°C) *^e^*
(iC4 + CP)	−38	−15	−91	−102	272	310
(iC4 + C18)	−7	+3	−18	+3	415	469
(CP + C18)	−3	+3	+18	+6	438	476
iC4&CP	−54	−22	−117	−110		
iC4&C18	−76	−8	−151	−99		
CP&C18	−14	−2	−42	−19		

*^a^* 40 wt % heterogeneous POSS and mixture with 20 wt % each POSS. *^b^* Compared with 406 °C. *^c^* Compared with 429 °C. *^d^* Compared with 388 °C. *^e^* Compared with 466 °C.

## Supplementary Materials

The following are available online at http://www.mdpi.com/2073-4360/11/1/44/s1.
