# Supplementary material for: Optical, Electrical and Thermal Properties of Organic–Inorganic Hybrids with Conjugated Polymers Based on POSS Having Heterogeneous Substituents
Source: Polymers (Basel). 2018 Dec 29;11(1):44. doi: 10.3390/polym11010044 (PMC6401744; doi:10.3390/polym11010044)
Supplement: Supplementary file 1 [file polymers-11-00044-s001.pdf]

Supporting Information

Optical, Electrical and Thermal Properties of Organic−Inorganic Hybrids with Conjugated Polymers Based on POSS Having Heterogeneous Substituents

Kazunari Ueda[1,2], Kazuo Tanaka*[1], Yoshiki Chujo[1]

[1]Department of Polymer Chemistry, Graduate School of Engineering, Kyoto University, Katsura, Nishikyo-ku, Kyoto 615-8510, Japan

[2]Matsumoto Yushi-Seiyaku Co., Ltd., 2-1-3, Shibukawa-cho, Yao-City, Osaka 581-0075, Japan

Phone: +81-75-383-2604, Fax: +81-75-383-2605

*To whom correspondence should be addressed: tanaka@poly.synchem.kyoto-u.ac.jp

Experimental Section

General. ^{1}H NMR and ^{13}C NMR spectra were measured with a JEOL EX-400 (400 MHz for ^{1}H and 100 MHz for ^{13}C) spectrometer. Coupling constants (J value) are reported in hertz. The chemical shifts are expressed in ppm downfield from tetramethylsilane using residual chloroform (δ = 7.24 in ^{1}H NMR, δ = 77.0 in ^{13}C NMR) as an internal standard. The number-average molecular weight (M_n) and the molecular weight distribution [weight-average molecular weight/number-average molecular weight (M_w/M_n)] values of all polymers were estimated by the size-exclusion chromatography (SEC) with a TOSOH G3000HXI system equipped with three consecutive polystyrene gel columns (TOSOH gels: α-4000, α-3000, and α-2500) and ultraviolet detector at 40 °C. The system was operated at a flow rate of 1.0 mL/min, with chloroform as an eluent. Polystyrene standards were employed for the calibration. UV–vis spectra were recorded on a Shimadzu UV-3600 spectrophotometer. Emission spectra were recorded on a HORIBA JOBIN YVON Fluoromax-4 spectrofluorometer, and absolute quantum yields were determined by the integrating sphere method on the HORIBA JOBIN YVON Fluoromax-4 spectrofluorometer. Differential scanning calorimetry (DSC) thermograms were carried out on a SII DSC 6220 instrument by using ~10 mg of exactly weighed samples at heating rate of 10 °C/min. The glass transition temperatures (T_g) were evaluated from the second

monitoring curves after annealing at 100 °C for 10 min, followed by cooling to 30 °C. Thermogravimetric analysis (TGA) was performed on an EXSTAR TG/DTA 6220, Seiko Instrument, Inc., with the heating rate of 10 °C/min up to 500 °C under nitrogen atmosphere. Residual chloroform was removed by keeping in a vacuum oven at 60 °C for 1 h before the TGA measurements. Aluminum electrodes were then deposited by thermal evaporation using vacuum evaporation system E-250DE, Shimadzu Emit Co., Ltd.. The time-of-flight (TOF) measurement to evaluate carrier mobilities was performed on TOF-401, Sumitomo Heavy Industries, Ltd., including N_2 laser system (KEC-160, Usho Optical Systems Co., Ltd.) and oscilloscope for recording the transient current (TDS 3052C, Tktronix, Inc.). Polymers and POSS derivatives including octa-substituted and heterogeneous-modified POSS were prepared according to the previous works.[1,2]

Synthesis of POSS fillers. The selected two kinds of trialkoxysilanes (isobutyltrimethoxysilane, 1.2 mL, 6.2 mmol; cyclopentyltriethoxysilane, 1.6 mL, 6.2 mmol; octadecyltriethoxysilane, 3.0 mL, 6.2 mmol) were dissolved in acetone (50 mL), and then tetrabutylammonium fluoride (7.0 mL of 1 M in THF) was added. The mixture was stirred at room temperature for 1 d, and white precipitation was generated. After filtration and drying *in vacuo*, the product was obtained as a white solid ((iC4+CP)POSS,

76%; (iC4+C18)POSS, 60%; (CP+C18)POSS, 62%). (iC4+CP)POSS: ^{1}H NMR ($CDCl_3$, 400 MHz) δ 1.85, 1.74, 1.53, 0.96, 0.60; ^{13}C NMR ($CDCl_3$, 400 MHz) δ 27.3, 27.0, 25.8, 23.9, 22.6, 22.3; ^{29}Si NMR ($CDCl_3$, 400 MHz) δ –66.52, –66.76, –67.00, –67.32, –67.62, –67.91. (iC4+C18)POSS: ^{1}H NMR ($CDCl_3$, 400 MHz) δ 1.85, 1.26, 0.96, 0.88, 0.58; ^{13}C NMR ($CDCl_3$, 400 MHz) δ 33.0, 31.9, 29.8, 29.4, 25.8, 23.9, 23.1, 22.7, 14.1; ^{29}Si NMR ($CDCl_3$, 400 MHz) δ –66.62, –66.73, –68.67, –68.84. (CP+C18)POSS: ^{1}H NMR ($CDCl_3$, 400 MHz) δ 1.74, 1.53, 1.26, 1.02, 0.88, 0.56; ^{13}C NMR ($CDCl_3$, 400 MHz) δ 33.0, 32.0, 29.8, 29.4, 27.5, 27.0, 24.3, 23.1, 22.7, 22.3, 19.9, 14.1; ^{29}Si NMR ($CDCl_3$, 400 MHz) δ –66.47, –68.55.

Preparation of hybrids. The mixtures (10 mL) containing 10 mg of the polymers and various amounts of POSS fillers in chloroform were stirred at room temperature for 3 h. The film samples were fabricated by dropping the mixture on the quartz plate (1 cm × 5 cm). Spin coating (1000 rpm) was performed with a MIKASA Spin Coater 1H-D7 (MIKASA Co. Ltd., Tokyo, Japan). The film samples were dried for 30 min under ambient condition. The resulting films on the quartz plate were used directly for the following measurements.

Preparation of the hybrids for the TOF experiment. The mixture (10 mL) containing 10 mg of the polymers and various amounts of POSS fillers in chloroform was stirred at room temperature for 3 h. For electrical measurement, the polymer solution was dropped on the etched indium tin oxide (ITO) coated glass substrates. The film sample was dried for 30 min under ambient condition. Aluminum electrodes were then deposited by thermal evaporation (at 1.3×10^{-3} Pa) through a mask onto the polymer film. The resulting sample with the sandwich structure (about 9 mm^2) and the electrode overlap active area was used directly for the electrical measurements.

Determination of carrier mobilities of the polymer composites.

The TOF measurement to evaluate carrier mobilities was performed under air. The TOF experiment involves photogeneration of charge carriers by illumination of a sample between two electrodes. The drift of the carriers under a bias to the collecting electrode results in a time-dependent current. Mobilities (μ) of the films can be described using the thickness of the film (d, about 1 μm), the external applied field (E, $1–5\times10^5$ V/cm), the transit time (t_T)

$$\mu = \frac{d}{Et_T} \qquad (1)$$

References

1. Tanaka, K.; Adachi, S.; Chujo, Y. *J. Polym. Sci. Part A: Polym. Chem*. **2009**, *47*, 5690−5697.

2. Ueda, K.; Tanaka, K.; Chujo, Y. *Bull. Chem. Soc. Jpn.* **2017**, *90*, 205−209.

Table S1. Polymer properties of PF and P3HT used in this study

	M_w	M_n	M_w/M_n
PF	120,000	35,000	3.6
P3HT	110,000	38,000	2.9

Table S2. Thermal properties of POSS

POSS	T_{d5} (°C)	T_{d20} (°C)
iC4	221	249
CP	330	378
C18	281	465

Table S3. Glass transition temperatures (T_gs) of the hybrids with POSS[a]

	PF	P3HT
	T_{d5} (°C)	T_{d5} (°C)
polymer	40	n.d.[b]
(iC4+CP)	40	n.d.[b]
(iC4+C18)	38	39
(CP+C18)	39	39
iC4&CP	40	42
iC4&C18	44	40
CP&C18	41	43

[a]40 wt% heterogeneous POSS and mixture with 20 wt% each POSS.

[b]Not detectable.

Table S4. Carrier-migration ability of the hybrids containing 40 wt% POSS

	Mobility (10^{-3} $cm^2/V{\cdot}s$)				
Bias voltage (V)	10	20	30	40	50
Applied field $(V/cm)^{1/2}$	316	447	548	632	707
PF	4.6	2.0	1.6	1.4	1.1
(iC4+CP)POSS	2.8	1.4	0.92	0.52	0.52
(iC4+C18)POSS	2.8	1.4	0.89	0.54	0.54
(CP+C18)POSS	2.8	1.4	0.89	0.53	0.53
iC4&CP	2.8	1.4	0.91	0.67	0.51
iC4&C18	2.8	1.4	0.88	0.65	0.53
CP&C18	3.0	1.3	0.80	0.71	0.55
P3HT	1.2	0.42	0.31	0.24	0.24
(iC4+CP)POSS	0.90	0.30	0.15	0.12	0.12
(iC4+C18)POSS	0.85	0.25	0.15	0.10	0.10
(CP+C18)POSS	0.84	0.27	0.15	0.12	0.12
iC4&CP	0.82	0.28	0.15	0.12	−
iC4&C18	0.83	0.25	0.15	0.10	−
CP&C18	0.75	0.25	0.16	0.10	−

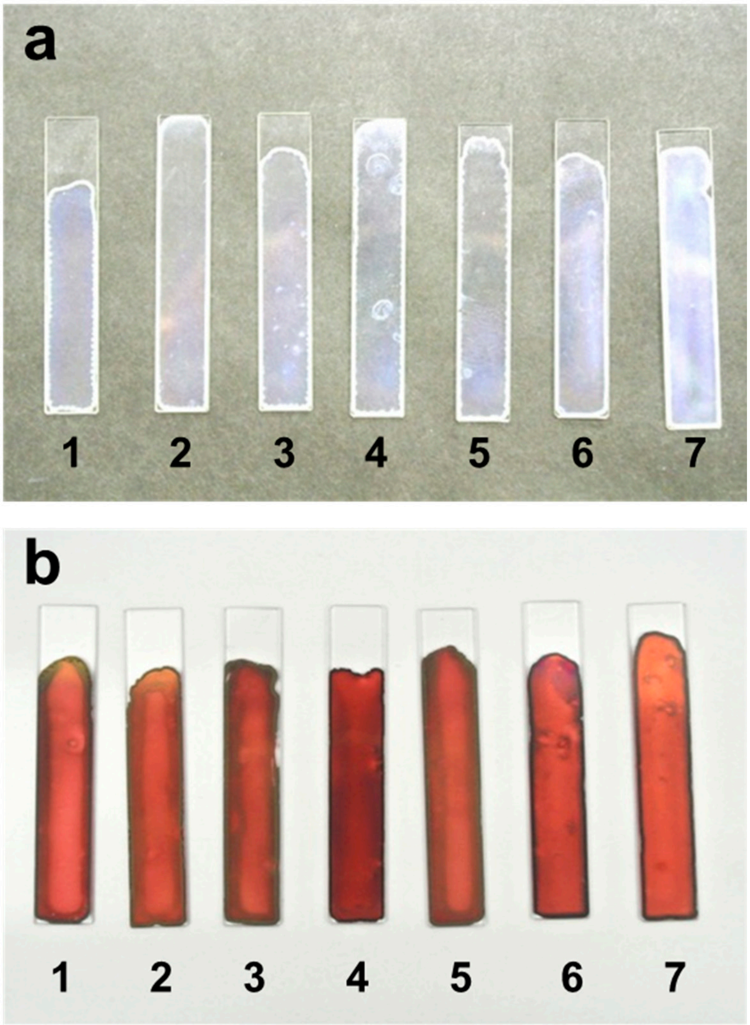

Figure S1. Appearances of the hybrid films with (a) PF and (b) P3HT in the 1) absence and presence of 2) 40 wt% (iC4+CP)POSS, 3) 40 wt% (iC4+C18)POSS, 4) 40 wt% (CP+C18)POSS, 5) 20 wt% iC4-POSS and 20 wt% CP-POSS, 6) 20 wt% iC4-POSS and 20 wt% C18-POSS and 7) 20 wt% CP-POSS and 20 wt% C18-POSS.

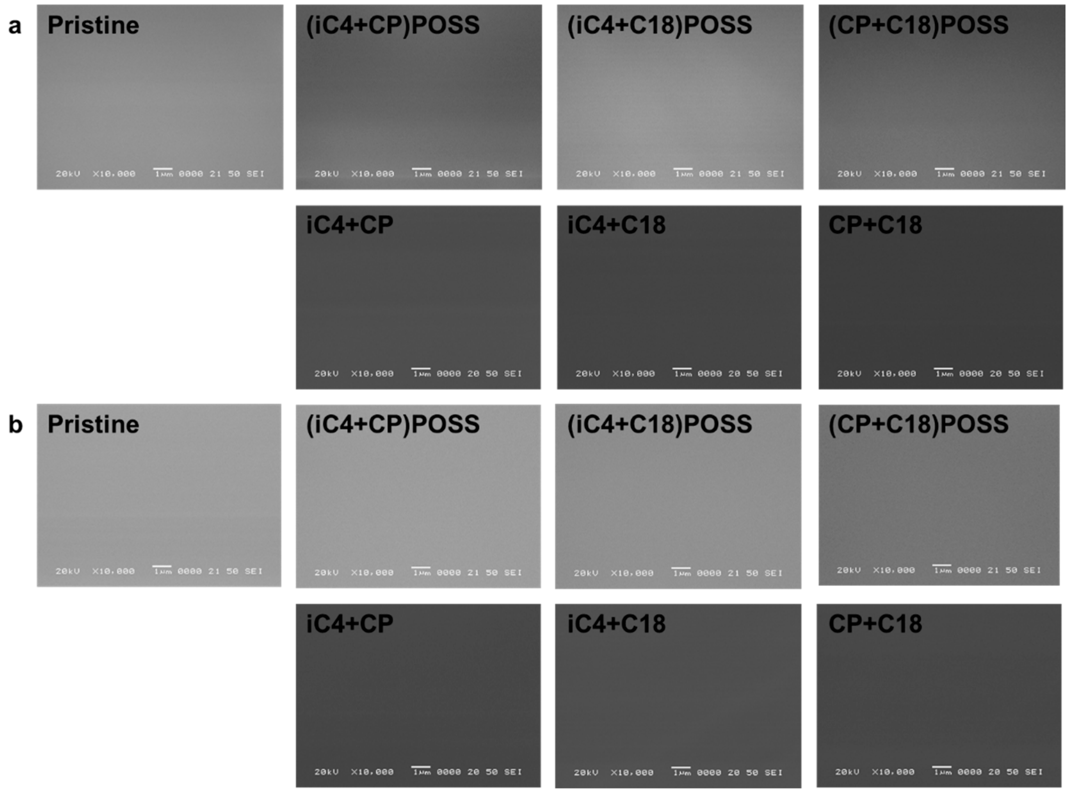

Figure S2. SEM images of the (a) PF and (b) P3HT samples. The scale bars represent 1 μm.

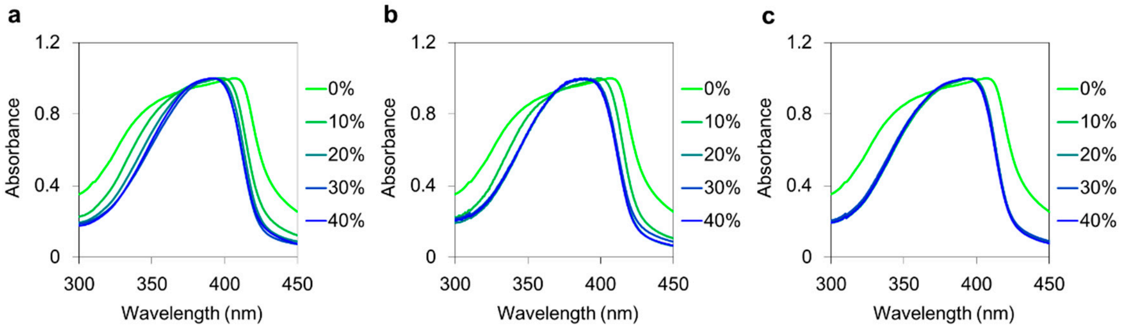

Figure S3. Normalized UV−vis absorption spectra of the PF hybrid films containing variable concentrations of (a) (iC4+CP)POSS, (b) (iC4+C18)POSS and (c) (CP+C18)POSS.

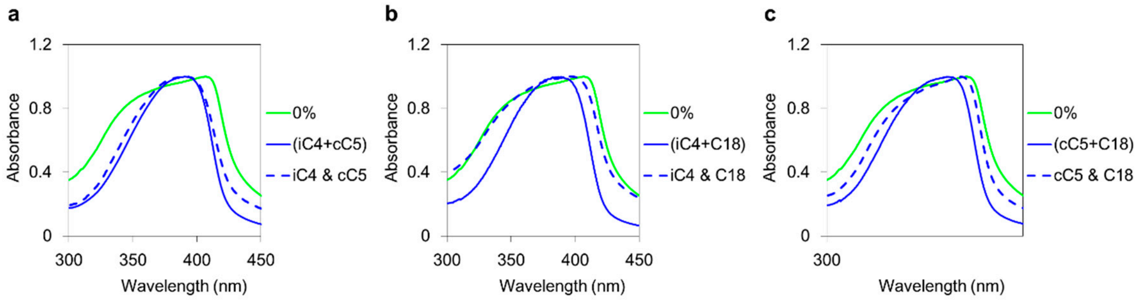

Figure S4. Normalized UV−vis absorption spectra of the PF hybrid films in the absence and presence of (a) 40 wt% (iC4+CP)POSS and the mixture with 20 wt% iC4-POSS and 20 wt% CP-POSS, (b) 40 wt% (iC4+C18)POSS and the mixture with 20 wt% iC4-POSS and 20 wt% C18-POSS and (c) 40 wt% (CP+C18)POSS and the mixture with 20 wt% CP-POSS and 20 wt% C18-POSS.

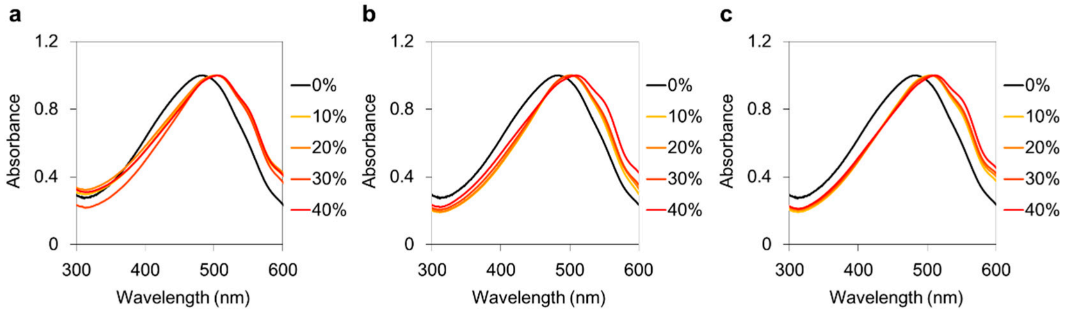

Figure S5. Normalized UV−vis absorption spectra of the P3HT hybrid films containing variable concentrations of (a) (iC4+CP)POSS, (b) (iC4+C18)POSS and (c) (CP+C18)POSS.

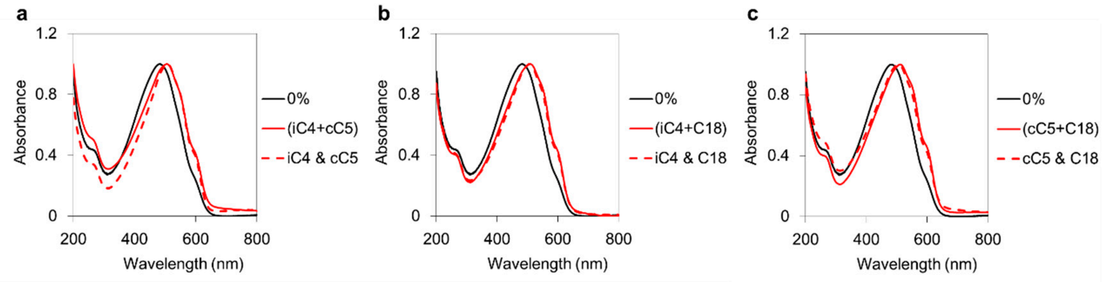

Figure S6. Normalized UV−vis absorption spectra of the P3HT hybrid films in the absence and presence of (a) 40 wt% (iC4+CP)POSS and the mixture with 20 wt% iC4-POSS and 20 wt% CP-POSS, (b) 40 wt% (iC4+C18)POSS and the mixture with 20 wt% iC4-POSS and 20 wt% C18-POSS and (c) 40 wt% (CP+C18)POSS and the mixture with 20 wt% CP-POSS and 20 wt% C18-POSS.

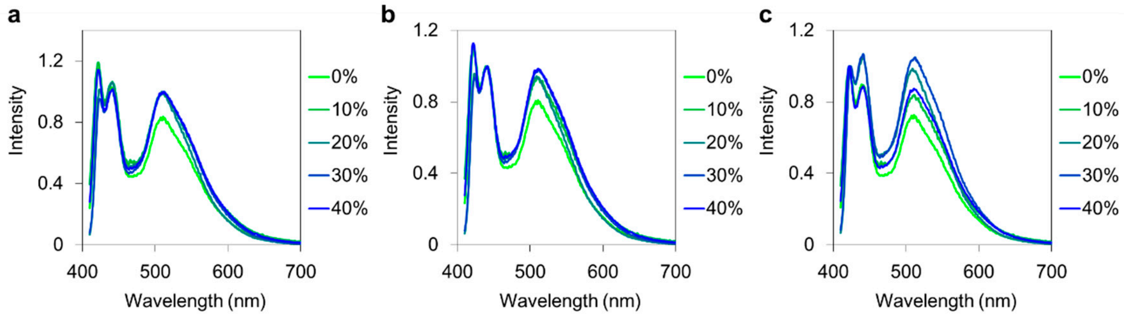

Figure S7. Normalized photoluminescence spectra of the PF hybrids containing variable concentrations of (a) (iC4+CP)POSS, (b) (iC4+C18)POSS and (c) (CP+C18)POSS.

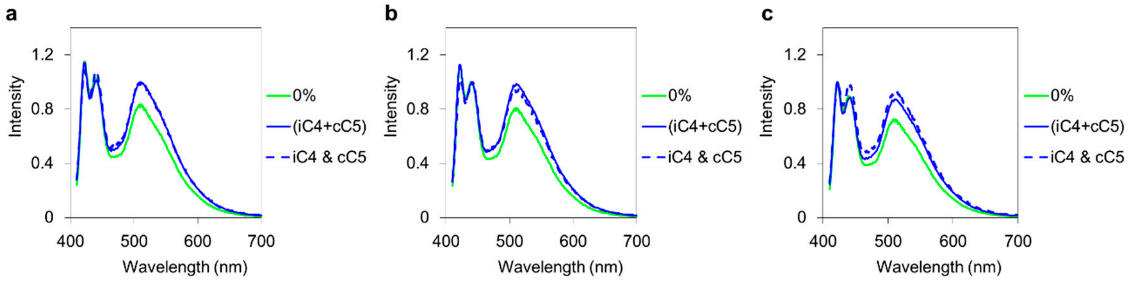

Figure S8. Normalized photoluminescence spectra of the PF hybrids in the absence and presence of (a) 40 wt% (iC4+CP)POSS and the mixture with 20 wt% iC4-POSS and 20 wt% CP-POSS, (b) 40 wt% (iC4+C18)POSS and the mixture with 20 wt% iC4-POSS and 20 wt% C18-POSS and (c) 40 wt% (CP+C18)POSS and the mixture with 20 wt% CP-POSS and 20 wt% C18-POSS.

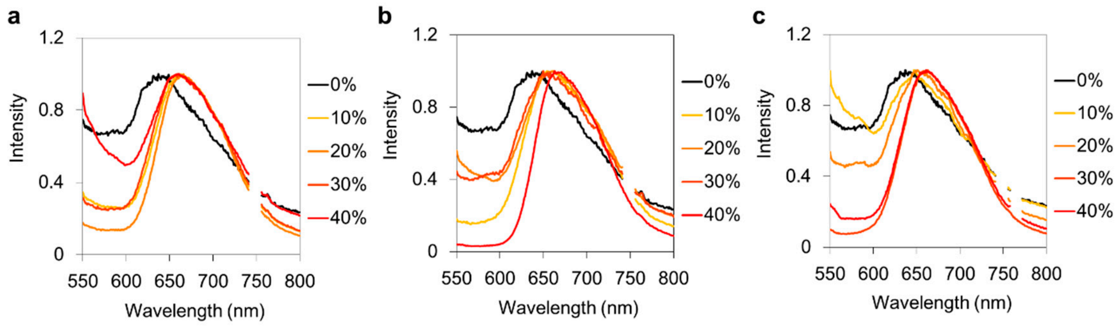

Figure S9. Normalized photoluminescence spectra of the P3HT hybrids containing variable concentrations of (a) (iC4+CP)POSS, (b) (iC4+C18)POSS and (c) (CP+C18)POSS.

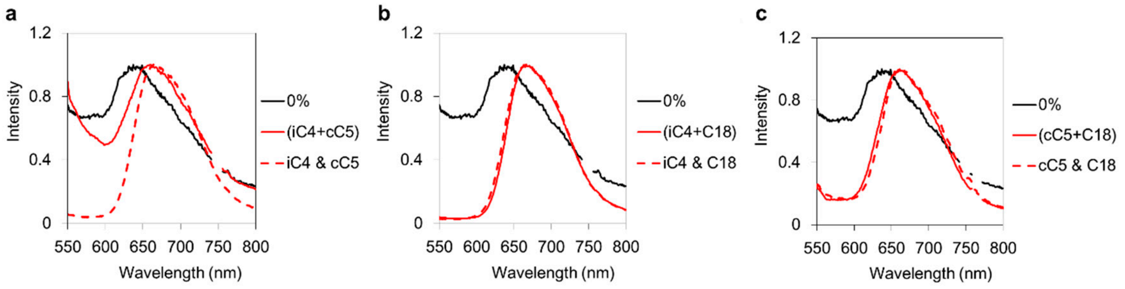

Figure S10. Normalized photoluminescence spectra of the P3HT hybrids in the absence and presence of (a) 40 wt% (iC4+CP)POSS and the mixture with 20 wt% iC4-POSS and 20 wt% CP-POSS, (b) 40 wt% (iC4+C18)POSS and the mixture with 20 wt% iC4-POSS and 20 wt% C18-POSS and (c) 40 wt% (CP+C18)POSS and the mixture with 20 wt% CP-POSS and 20 wt% C18-POSS.

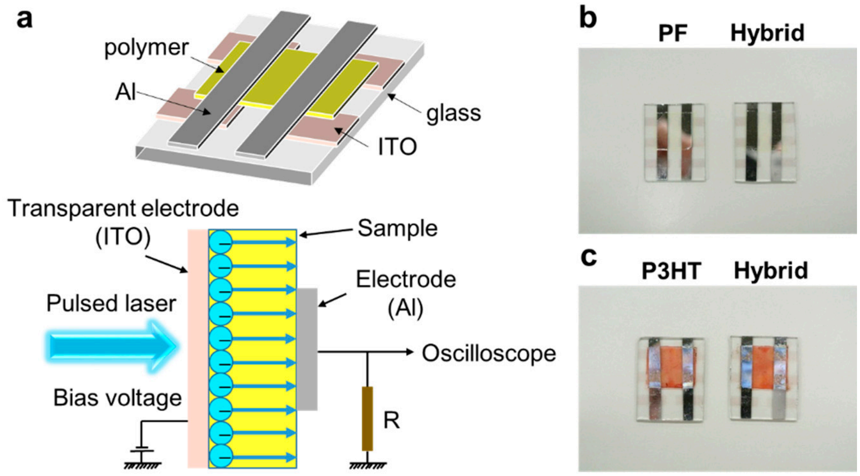

Figure S11. (a) Illustrated device structures for the TOF experiment and appearances of the film samples based on (b) PF and (c) P3HT.

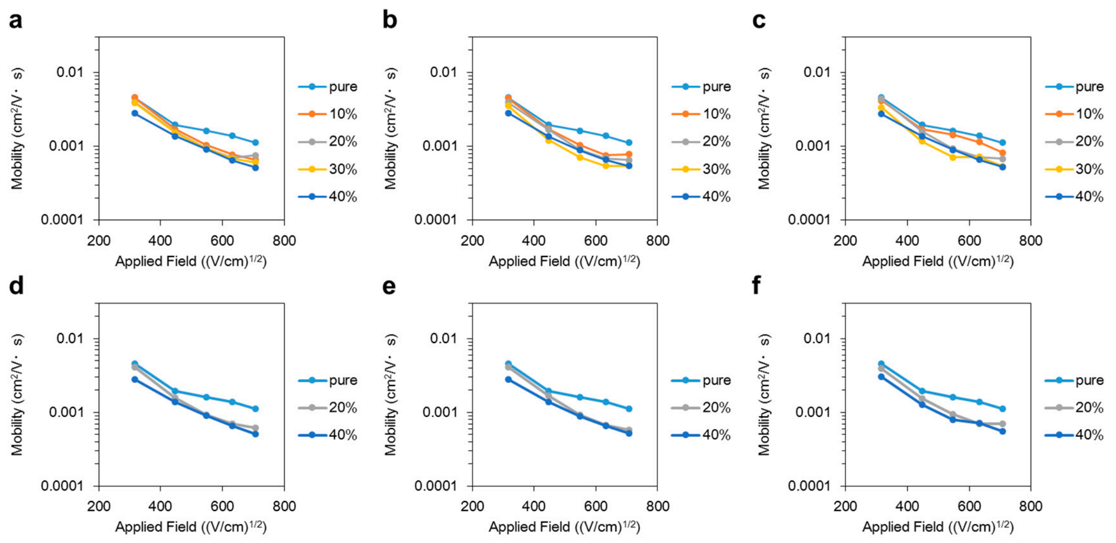

Figure S12. Carrier-mobility changes by variable applied fields with the PF samples containing (a) (iC4+CP)POSS, (b) (iC4+C18)POSS, (c) (CP+C18)POSS and the same amounts of (d) iC4-POSS and CP-POSS, (e) iC4-POSS and C18-POSS and (f) CP-POSS and C18-POSS.

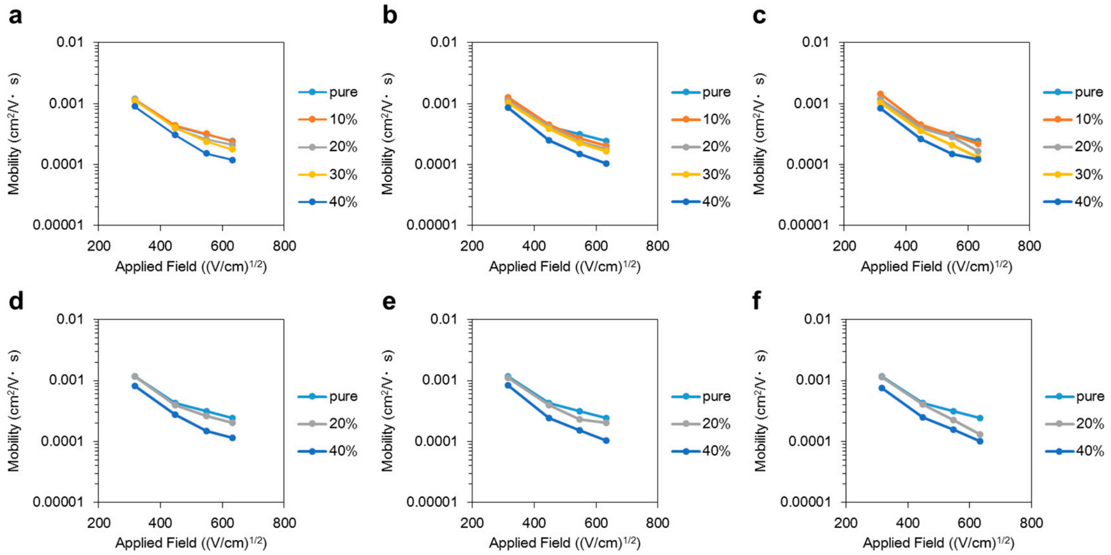

Figure S13. Carrier-mobility changes by variable applied fields with the P3HT samples containing (a) (iC4+CP)POSS, (b) (iC4+C18)POSS, (c) (CP+C18)POSS and the same amounts of (d) iC4-POSS and CP-POSS, (e) iC4-POSS and C18-POSS and (f) CP-POSS and C18-POSS.
